# Simple Parameters from Complete Blood Count Predict In-Hospital Mortality in COVID-19

**DOI:** 10.1155/2021/8863053

**Published:** 2021-05-13

**Authors:** Mattia Bellan, Danila Azzolina, Eyal Hayden, Gianluca Gaidano, Mario Pirisi, Antonio Acquaviva, Gianluca Aimaretti, Paolo Aluffi Valletti, Roberto Angilletta, Roberto Arioli, Gian Carlo Avanzi, Gianluca Avino, Piero Emilio Balbo, Giulia Baldon, Francesca Baorda, Emanuela Barbero, Alessio Baricich, Michela Barini, Francesco Barone-Adesi, Sofia Battistini, Michela Beltrame, Matteo Bertoli, Stephanie Bertolin, Marinella Bertolotti, Marta Betti, Flavio Bobbio, Paolo Boffano, Lucio Boglione, Silvio Borrè, Matteo Brucoli, Elisa Calzaducca, Edoardo Cammarata, Vincenzo Cantaluppi, Roberto Cantello, Andrea Capponi, Alessandro Carriero, Giuseppe Francesco Casciaro, Luigi Mario Castello, Federico Ceruti, Guido Chichino, Emilio Chirico, Carlo Cisari, Micol Giulia Cittone, Crizia Colombo, Cristoforo Comi, Eleonora Croce, Tommaso Daffara, Pietro Danna, Francesco Della Corte, Simona De Vecchi, Umberto Dianzani, Davide Di Benedetto, Elia Esposto, Fabrizio Faggiano, Zeno Falaschi, Daniela Ferrante, Alice Ferrero, Ileana Gagliardi, Alessandra Galbiati, Silvia Gallo, Pietro Luigi Garavelli, Clara Ada Gardino, Massimiliano Garzaro, Maria Luisa Gastaldello, Francesco Gavelli, Alessandra Gennari, Greta Maria Giacomini, Irene Giacone, Valentina Giai Via, Francesca Giolitti, Laura Cristina Gironi, Carla Gramaglia, Leonardo Grisafi, Ilaria Inserra, Marco Invernizzi, Marco Krengli, Emanuela Labella, Irene Cecilia Landi, Raffaella Landi, Ilaria Leone, Veronica Lio, Luca Lorenzini, Antonio Maconi, Mario Malerba, Giulia Francesca Manfredi, Maria Martelli, Letizia Marzari, Paolo Marzullo, Marco Mennuni, Claudia Montabone, Umberto Morosini, Marco Mussa, Ilaria Nerici, Alessandro Nuzzo, Carlo Olivieri, Samuel Alberto Padelli, Massimiliano Panella, Andrea Parisini, Alessio Paschè, Filippo Patrucco, Giuseppe Patti, Alberto Pau, Anita Rebecca Pedrinelli, Ilaria Percivale, Luca Ragazzoni, Roberta Re, Cristina Rigamonti, Eleonora Rizzi, Andrea Rognoni, Annalisa Roveta, Luigia Salamina, Matteo Santagostino, Massimo Saraceno, Paola Savoia, Marco Sciarra, Andrea Schimmenti, Lorenza Scotti, Enrico Spinoni, Carlo Smirne, Vanessa Tarantino, Paolo Amedeo Tillio, Stelvio Tonello, Rosanna Vaschetto, Veronica Vassia, Domenico Zagaria, Elisa Zavattaro, Patrizia Zeppegno, Francesca Zottarelli, Pier Paolo Sainaghi

**Affiliations:** ^1^Università del Piemonte Orientale UPO, Novara, Italy; ^2^“AOU Maggiore della Carità”, Novara, Italy; ^3^Azienda Ospedaliera SS. Antonio e Biagio e Cesare Arrigo, Alessandria, Italy; ^4^“Sant'Andrea” Hospital, Vercelli, Italy

## Abstract

**Introduction:**

The clinical course of Coronavirus Disease 2019 (COVID-19) is highly heterogenous, ranging from asymptomatic to fatal forms. The identification of clinical and laboratory predictors of poor prognosis may assist clinicians in monitoring strategies and therapeutic decisions.

**Materials and Methods:**

In this study, we retrospectively assessed the prognostic value of a simple tool, the complete blood count, on a cohort of 664 patients (*F* 260; 39%, median age 70 (56-81) years) hospitalized for COVID-19 in Northern Italy. We collected demographic data along with complete blood cell count; moreover, the outcome of the hospital in-stay was recorded.

**Results:**

At data cut-off, 221/664 patients (33.3%) had died and 453/664 (66.7%) had been discharged. Red cell distribution width (RDW) (*χ*^2^ 10.4; *p* < 0.001), neutrophil-to-lymphocyte (NL) ratio (*χ*^2^ 7.6; *p* = 0.006), and platelet count (*χ*^2^ 5.39; *p* = 0.02), along with age (*χ*^2^ 87.6; *p* < 0.001) and gender (*χ*^2^ 17.3; *p* < 0.001), accurately predicted in-hospital mortality. Hemoglobin levels were not associated with mortality. We also identified the best cut-off for mortality prediction: a NL ratio > 4.68 was characterized by an odds ratio for in-hospital mortality (OR) = 3.40 (2.40-4.82), while the OR for a RDW > 13.7% was 4.09 (2.87-5.83); a platelet count > 166,000/*μ*L was, conversely, protective (OR: 0.45 (0.32-0.63)).

**Conclusion:**

Our findings arise the opportunity of stratifying COVID-19 severity according to simple lab parameters, which may drive clinical decisions about monitoring and treatment.

## 1. Introduction

The global spread of severe acute respiratory syndrome coronavirus 2 (SARS-CoV-2) generated a pandemic outbreak in late 2019-early 2020, which severely impacted on care delivery worldwide. The clinical picture caused by the abovementioned viral infection has been called Coronavirus disease 2019 (COVID-19): its clinical manifestations encompass a wide range of entities, from a mild flu-like illness to life-threatening forms [1]. Despite differences among countries, the overall in-hospital mortality is high, being reported up to 30% [2]. The reasons underlying such a different clinical behavior are still unknown; thus, it is difficult to predict who will develop a severe clinical picture and who, conversely, will show a mild disease.

Thus, the identification of predictors of mortality might be valuable to adjust clinicians' approach to monitoring and therapy. Among clinical parameters, proposed predictors of poor prognosis include age, comorbidities, and male gender [3, 4]. Moreover, some laboratory variables have been associated to mortality in different cohorts [5, 6]; in a recent meta-analysis, elevated cardiac troponin, C-reactive protein, interleukin-6, D-dimer, creatinine, liver function tests and decreased levels of albumin turned out to predict mortality among COVID-19 hospitalized patients [7]. Furthermore, preliminary reports suggest that blood cell count may be particularly relevant [8, 9]. Blood cell count is a simple, highly informative, and universally available exam. The validation of these early reports may give to clinicians a further valuable tool for clinical risk stratification. On these grounds, here we aimed to verify whether simple data retrievable from blood cell count may predict the outcome in patients admitted to the hospital because of COVID-19.

## 2. Methods

### 2.1. Study Population

The study was conducted at the “Maggiore della Carità” University Hospital in Novara, Northern Italy.

Scanning the hospital administrative database, we retrieved all consecutive patients older than 18 years of age, admitted to the hospital after emergency room evaluation, with a confirmed diagnosis of SARS-CoV-2 infection by reverse-transcriptase polymerase chain reaction (RT-PCR) of a nasopharyngeal swab, between 1^st^ March 2020 and 28^th^ April 2020. We identified a total of 763 patients.

We revised data available from central lab software to retrieve the complete blood cell count at hospital admission, along with gender and age. We also recorded the outcome of in-hospital stay (discharged or deceased).

The study protocol was approved by the Institutional Review Board (Comitato Etico Interaziendale Novara; IRB code CE 97/20) and conducted in strict accordance with the principles of the Declaration of Helsinki. Prospective informed consent was waived by competent authorities due to the retrospective nature of the study and the use of pseudonymized data.

### 2.2. Statistical Analysis

Data were summarized according to groups as median and (25th-75th percentile) and analyzed using the Wilcoxon test. Categorical variables, whenever dichotomous or nominal, were reported as frequencies and percentages and analyzed through the Chi-square test.

The optimal cut-off value for each predictor has been computed considering as criterion the sum of sensitivity and specificity maximization [10]. The cut-off values have been considered to dichotomize the predictors; the area under curve (AUC) values in discriminating the in-hospital mortality have been reported.

Univariate analysis and multivariate logistic regression were carried out to quantify the effects of the dichotomized covariates on in-hospital mortality. We included in the multivariate models the following covariates: age, gender, and laboratory parameters derived from complete blood count.

The .632 bootstrap (1000 resamples) validation procedure was carried out to evaluate the predictive logistic regression model performance reporting the Harrell-C statistics corrected for overoptimism [11].

The *p* values have been adjusted via Holm multiple testing procedure.

Statistical analyses were conducted using R 3.5.2 with the rms packages [12]. The threshold of statistical significance was 0.05 for all tests used (two tailed).

## 3. Results

At data cut-off, 664 (260 females, 39%; median age 70 (56-81) years) patients completed their hospital in-stay: 221/664 patients (33.3%) had died and 453/664 (66.7%) had been discharged. The median time to death was 6 days [2–10], while the median time to discharge was 8 days [5–14] (*p* < 0.001).

Table 1 reports the main features of the study population. The proportion of males was higher among those who died in-hospital; moreover, the age was significantly higher in this group of subjects, who also showed increased white blood cells (WBC), absolute neutrophil count (ANC), neutrophil-to-lymphocyte ratio (NL ratio), eosinophil count, mean corpuscular hemoglobin (MCH), mean corpuscular volume (MCV), and red cell distribution width (RDW). Conversely, survivors had significantly higher platelet count, hemoglobin concentration, and absolute lymphocyte count (ALC).

We ran a first multivariate model to test the predictive role of all the parameters derived from complete blood count. The results are shown in Table 2. Along with age and gender, RDW was the strongest independent predictor of mortality; ANC was still significantly and independently predictive of in-hospital mortality, while the contribution given by WBC, ALC, and platelet count was weaker. Predictably, the effect of NL ratio was blunted by the inclusion of ANC and ALC into the model.

On the basis of the first model, we ran a further multivariate analysis, including age, gender, platelet count, NL ratio (used as a summary measure for white blood cell count and differential), RDW, and hemoglobin. RDW (*χ*^2^ 10.40; *p* < 0.001), NL ratio (*χ*^2^ 7.59; *p* = 0.006), and platelet count (*χ*^2^ 5.39; *p* = 0.02), along with age (*χ*^2^ 87.6; *p* < 0.001) and gender (*χ*^2^ 17.3; *p* < 0.001, confirmed their independent predictive role. Conversely, hemoglobin (*χ*^2^ 2.28; *p* = 0.131) was not associated with outcome.

We further tried to identify the best performing cut-off for these parameters; in Table 3, we reported our results, with the respective area under the curve (AUC) and odds ratio.

On these bases, we built a final multivariate model which confirmed that age > 74 years (*χ*^2^ 67.1; *p* < 0.001), male gender (*χ*^2^ 14.9; *p* < 0.001), platelets < 166,000 (*χ*^2^ 22.3; *p* < 0.0001), NL ratio > 4.68 (*χ*^2^ 21.4; *p* < 0.0001), and RDW > 13.7% (*χ*^2^ 25.2; *p* < 0.001) were independent predictors of in-hospital mortality. Once again, hemoglobin (*χ*^2^ 0.03; *p* = 0.85) was not associated to in-hospital mortality.

## 4. Discussion

In the present paper, we investigated a cohort of hospitalized patients in Northern Italy with a defined diagnosis of COVID-19, showing that simple complete blood count parameters obtained at hospital admission accurately predict in-hospital mortality. A first finding deserving a comment is the observed mortality which, in our cohort, was very high. More than 30% of patients died during hospital stay. This is in line with previous observations from other groups [13–15], although data about in-hospital mortality widely vary, with case fatality rates much lower in the first reported cohorts from China [16, 17]. The reasons of this discrepancy are still largely unknown; however, both the phase of the epidemic curve and the geographic region have an impact in determining a very different prognosis in COVID-19 patients. Our study is adequately powered to compensate for variations in the mortality rate: for example, with a NL ratio above 7 in 25% of discharged patients and 50% of deceased patients, the estimated power is 0.9999 for at a mortality rate of 0.33 (33%) and remains >0.80 for all populations with mortality rate above 0.06 (6%).Thus, our data strongly support the idea that complete blood count, a routine test for most patients admitted to hospital, might be highly informative with regard to prognosis of COVID-19 patients admitted to hospital. This is in line with different studies reporting a potential role for blood count in the prediction of mortality in the context of Emergency Medicine, cardiovascular and cerebrovascular diseases [18–21].

More specifically, we identified three main predictors of in-hospital death: NL ratio, platelet count, and RDW.

NL ratio has already been described to be strongly associated with COVID-19 prognosis [22–24]. The ratio magnifies the strength of the association of both components, neutrophil count and lymphocyte count, in predicting the outcome for COVID-19 patients. Whether neutrophilia is just a biomarker of disease severity or has a pathogenetic role is unclear. However it is worth mentioning that COVID-19 mortality seems to be frequently related to the development of thromboembolic complications and systemic inflammation [25, 26]. Neutrophilia is a known marker of venous thrombosis, possibly contributing by driving a necroinflammatory response [27]. Furthermore, neutrophils play their protective role not only through phagocytosis but also exploiting neutrophil extracellular trap (NET) formation [28]. Although NETs are beneficial in the host defense against pathogens, sustained NET formation may drive collateral damage in different human conditions, including viral infections [29]. It has been postulated that, in COVID-19, the inappropriate NET production might be crucial in the development of the “cytokine storm” responsible of acute respiratory distress syndrome (ARDS), severe inflammatory response syndrome (SIRS), and sepsis [27]. With respect to lymphocytes, it has been suggested that SARS-CoV-2 infection may primarily affect T lymphocytes [30] and that lymphopenia in severe patients is mainly related to a decreased ALC, especially CD8+ T cells [31]. Since T cells are important for dampening overactive innate immune responses during viral infection, their loss during SARS-CoV-2 infection may result in a more severe inflammatory responses [32].

We suggest to use NL ratio, in this context, being its prognostic value higher, as shown by our data. In fact, NL ratio magnifies the predictive potential of neutrophilia and lymphopenia, being 4.68 the best prognostic cut-off.

With respect to platelets, some authors suggested that thrombocytopenia may be a negative prognostic factor. In a recent meta-analysis on 31 studies including 7163 patients, a lower platelet count predicted severe COVID-19 [33]; the mechanism by which SARS-CoV-2 leads to platelet consumption, especially in case of severe disease, is unknown. A possible explanation is that the lung damage might lead to platelet activation and aggregation, finally causing thrombocytopenia [34]. A limitation of our study is that we did not exclude patients affected by liver cirrhosis, which is tipically associated to thrombocytopenia; this might have partially biased our finding [35].

Finally, we demonstrated that RDW is a very strong predictor of mortality. According to our data, RDW is characterized by the highest *χ*^2^ and OR for in-hospital mortality among all the variables considered. The clinical value of RDW is often neglected; in the last few years, it has been recognized as a common prognostic predictor in many human diseases: acute kidney injury, cancer, acute pancreatitis, and respiratory failure [36–40]. The predictive significance of RDW in the context of COVID-19 might reflect the effect on erythropoiesis of respiratory failure and systemic inflammation [41, 42].

Our study has some limitations, due to the context in which we were forced to act by the pandemic; in particular, the observational nature of the study limited data availability and did not allow us to correct for potential confounders. Despite these limitations, our findings may raise the opportunity of stratifying disease severity according to simple lab parameters, which may drive clinical decisions about monitoring and treatment.

## 5. Conclusions

In the present study, we demonstrated that some simple parameters derived from complete blood cell count have prognostic implications in the course of COVID-19, being able to identify those patients at higher risk of in-hospital mortality. The confirmation of these preliminary observations may make available to clinicians novel useful tools for prognostic stratification.

## Figures and Tables

**Table 1:** Clinical features of the study population. The main general features of the study population are reported. Categorical variables, whenever dichotomous or nominal, were reported as frequencies and percentages and analyzed through the Chi-square test.

	Total (*N* 664)	Discharged (*N* 453)	Dead (*N* 211)	*p*
Age (years)	70 (56-81)	63 (52-75)	80 (73-87)	<0.0001
Gender (M/F)	404 (61)/260 (39)	260 (57)/193 (43)	144 (68)/67 (32)	0.008
WBC (×10^3^/*μ*L)	7.3 (5.2-10.9)	6.8 (5.1-9.7)	8.4 (5.8-12.0)	<0.0001
Neutrophils (×10^3^/*μ*L)	5.3 (3.5-8.6)	4.8 (3.4-7.6)	6.7 (4.1-9.6)	<0.0001
Lymphocytes (×10^3^/*μ*L)	1.07 (0.76-1.54)	1.15 (0.82-1.56)	0.92 (0.66-1.92)	<0.0001
Eosinophils (×10^3^/*μ*L)	0.01 (0.00-0.03)	0.01 (0.00-0.04)	0.00 (0.00-0.01)	<0.0001
NL ratio	4.7 (2.8-9.1)	4.0 (2.5-7.0)	7.0 (3.8-13.2)	<0.0001
Platelets (×10^3^/*μ*L)	195 (149-249)	198 (157-250)	168 (129-243)	<0.0001
Hb (gr/dL)	13.4 (11.8-14.7)	13.7 (12.1-14.8)	12.9 (11.4-14.4)	0.08
MCH (pg)	29.5 (28.2-30.7)	29.3 (28.3-30.5)	29.8 (28.0-31.1)	0.04
MCV (fL)	89.5 (85.7-93.4)	88.8 (85.1-91.9)	91.3 (86.9-94.6)	<0.0001
RDW (%)	13.7 (12.9-15.1)	13.3 (12.7-14.4)	14.5 (13.5-15.9)	<0.0001

Comparison between survivors and dead has been performed by a nonparametric Wilcoxon test. For abbreviation: M: males; F: females; WBC: white blood cells; NL ratio: neutrophil-to-lymphocyte ratio; MCH: mean cell hemoglobin; MCV: mean corpuscular volume; RDW: red cell distribution width.

**Table 2:** Multivariable model. The table shows a multivariable model including all blood count-derived variables.

	*χ* ^2^	*p*
Age (years)	77.04	<0.0001
Gender (M/F)	17.3	<0.0001
WBC (×10^3^/*μ*L)	3.63	0.06
Neutrophils (×10^3^/*μ*L)	4.25	0.04
Lymphocytes (×10^3^/*μ*L)	3.78	0.05
Eosinophils (×10^3^/*μ*L)	1.71	0.19
NL ratio	0.48	0.49
Platelets (×10^3^/*μ*L)	3.74	0.05
Hb (gr/dL)	0.37	0.54
MCH (pg)	0.56	0.45
MCV (fL)	0.32	0.57
RDW (%)	12.74	<0.0001

For abbreviation: M: males; F: females; WBC: white blood cells; NL ratio: neutrophil-to-lymphocyte ratio; MCH: mean cell hemoglobin; MCV: mean corpuscular volume; RDW: red cell distribution width.

**Table 3:** Univariable analysis. The optimal cut-off value for each predictor has been reported together with AUC (area under the curve) values. The univariable logistic regression OR (odds ratio) together with 95% confidence interval (CI) have been also reported.

	Cut-off	AUC	OR (CI95%)	*p*
Age (years)	74	0.786	6.90 (4.80-9.93)	<0.0001
WBC (×10^3^/*μ*L)	8,360	0.587	1.98 (1.42-2.77)	<0.0001
Neutrophils (×10^3^/*μ*L)	6,310	0.617	2.56 (1.83-3.59)	<0.0001
Lymphocytes (×10^3^/*μ*L)	1,130	0.618	0.42 (0.30-0.60)	<0.0001
Eosinophils (×10^3^/*μ*L)	0,100	0.587	0.57 (0.41-0.80)	0.0001
NL ratio	4.68	0.661	3.40 (2.40-4.82)	<0.0001
Platelets (×10^3^/*μ*L)	166	0.582	0.45 (0.32-0.63)	<0.0001
Hb (gr/dL)	13.0	0.577	0.60 (0.39-0.75)	0.0002
MCH (pg)	30.8	0.549	1.84 (1.26-2.68)	0.0001
MCV (fL)	90.6	0.602	2.51 (1.80-3.52)	<0.0001
RDW (%)	13.7	0.689	4.09 (2.87-5.83)	<0.0001

## Data Availability

Data are available upon reasonable request to the corresponding author.
